# Nutritional Outcomes Related to Household Food Insecurity among Mothers in Rural Malaysia

**DOI:** 10.3329/jhpn.v31i4.20031

**Published:** 2013-12

**Authors:** A.N. Ihab, A.J. Rohana, W.M. Wan Manan, W.N. Wan Suriati, M.S. Zalilah, A. Mohamed Rusli

**Affiliations:** ^1^Department of Community Medicine, School of Medical Sciences,; ^2^Program of Nutrition & Dietetics, School of Health Sciences, Health Campus, Universiti Sains Malaysia, Kubang Kerian, 16150 Kelantan, Malaysia; ^3^Program of Nutrition and Dietetics, Faculty of Medicine and Health Sciences, Universiti Putra Malaysia, Serdang 43400 Selangor, Malaysia

**Keywords:** Household food insecurity, Obesity, Overweight, Malaysia

## Abstract

During the past two decades, the rates of food insecurity and obesity have risen. Although a relationship between these two seemingly-paradoxical states has not been repeatedly seen in men, research suggests that a correlation between them exists in women. This study examines nutritional outcomes of household food insecurity among mothers in rural Malaysia. A cross-sectional survey of low-income households was conducted, and 223 households with mothers aged 18–55 years, who were non-lactating, non-pregnant, and had at least one child aged 2–12 years, were purposively selected. A questionnaire was administered that included the Radimer/Cornell Scale, items about sociodemographic characteristics, and anthropometric measurements. Of the households, 16.1% were food-secure whereas 83.9% experienced some kind of food insecurity: 29.6% of households were food-insecure, 19.3% contained individuals who were food-insecure, and 35.0% fell into the ‘child hunger’ category. The result reported that household-size, total monthly income, income per capita, and food expenditure were significant risk factors of household food insecurity. Although there was a high prevalence of overweight and obese mothers (52%) and 47.1% had at-risk waist-circumference (≥80 cm), no significant association was found between food insecurity, body mass index, and waist-circumference. In conclusion, the rates of household food insecurity and overweight and obesity were high in the study population, although they are looking paradoxical. Longitudinal studies with larger sample-sizes are recommended to further examine the relationship between food insecurity and obesity.

## INTRODUCTION

Food insecurity, as defined by the Life Sciences Research Office, exists “whenever the availability of nutritionally adequate and safe foods or the ability to acquire acceptable foods in socially acceptable ways is limited or uncertain” (1). An uncertain food supply, problems with food quantity and quality, running out of food, lacking money to buy food, skipping meals, and going hungry are all elements of the concept of food insecurity (2,3). Households suffering from food insecurity are more likely to have adults who have lower nutrient intakes (4,5), greater probabilities of having mental health problems (6), long-term physical health problems (7), higher levels of depression (8), higher levels of chronic disease (9), and lower scores on physical and mental health evaluations (10). Food-insecure seniors are more likely to have limitations in activities of daily living (11), being overweight and obese, especially among women from marginal and low-income households. Mothers who suffer chronic caloric or micronutrient deficiencies are more likely to have low-birthweight babies, subsequently passing their nutritional defect to the next generation (12). This consequence has a negative impact on the health of infants and toddlers (13,14). In Malaysia and other countries undergoing rapid transitions of various types in the Asian region, the prevalence of overweight and obesity is on the rise among both urban and rural populations (15-17). Obesity may also occur in households that are food-insecure as evidenced by the co-existence of underweight and overweight in the same poor households (18-20). In these countries, while positive associations between obesity and food insecurity with poverty have been well-documented and elucidated, the association between food insecurity and obesity and its plausible explanation remained elusive.

Campbell's conceptualization of household food insecurity included two sets of potential consequences of food insecurity. These include suboptimal nutritional status and non-nutritional outcomes, such as physical impairment, social isolation, and psychological instabilities (21). Although the association between household food insecurity and adverse health outcomes of adults has been established, its relationship with nutritional status has not been (22). This claim is based on previous studies (23-26). However, other scholars claim otherwise (27). In developed countries, household food insecurity has been associated with overweight and obesity of adults (17,23,25). In contrast, it has been associated with underweight among adults in other parts of the world (28,29). In other studies, food insecurity status was not associated with weight changes among mothers (30,31). These findings illustrate that the relationship between household food insecurity and nutritional status of adults is variable and differs from one region to another, depending on the context (32). The aims of this study were to determine the sociodemographic factors which related to household food insecurity in rural Malaysia and to examine whether household food insecurity is associated with maternal nutritional status in this population.

## MATERIALS AND METHODS

### Study location

Bachok is a district in Kelantan, Malaysia (Figure). It is located 25 km east of Kota Bharu. It borders Pasir Puteh to the south and Kota Bharu to the west. Mukims (smaller subdivisions) in Bachok district are: Tawang, Perupok, Repek, Telong, Gunung, Mahligai, Tanjong Pauh, Melawi, and Bekelam. Approximately 1,16,128 people live in Bachok. Malays are the predominant ethnic group in Bachok as in the rest of Kelantan, and Chinese and Siamese are the minority groups. Kelantan is considered to be one of the poorer states in the country. It has the highest rate of moderate undernutrition (24%) and severe undernutrition (5.9%) in Malaysia. It is also one of the top five states in Malaysia receiving food basket assistance for malnourished children from poor families as part of a poverty eradication programme since 1998 (33).

### Selection of subjects

In the Bachok district, 12 villages with Malay ethnic groups comprise the majority of the population. Based on information on population density from the District Office, eight of the largest villages were purposively selected for a cross-sectional study. The present research used the records of Social Welfare Department, which include all the recipients of monthly allowances in Bachok, Kelantan. Monthly welfare allowance is given to families earning below the government-defined Poverty Line Income (PLI) [based on Malaysian Ringgit (RM) 118.04 per capita Poverty Line Index**]** (34). The total number of recipient households was 3,635. The key informants (i.e. village head, welfare officer) contacted respondents receiving financial assistance to arrange for a meeting with the researchers at their homes. Respondents were mothers. Mothers were recruited because they were primarily responsible for food acquisition and preparation in the households. Enumerators visited the households to identify those eligible households with the following inclusion criteria: recipients of welfare assistance in Bachok district, presence of mothers aged 18 to 55 years, mothers who were neither pregnant nor lactating during the study period, households having children aged 2 to 12 years and living with the mother in the same household and signed the consent form. If there is more than one mother in the targeted household, only one mother who is responsible for food preparation was selected. Mothers and their children are involved in most household food security studies because the attitudes and practices of mothers may influence the eating habits of their children (35), and the health of mothers can be adversely affected by food scarcity and maternal hardship (10,36,37). The sample-size was calculated using the single proportion formula as follows (38):

**Fig.**
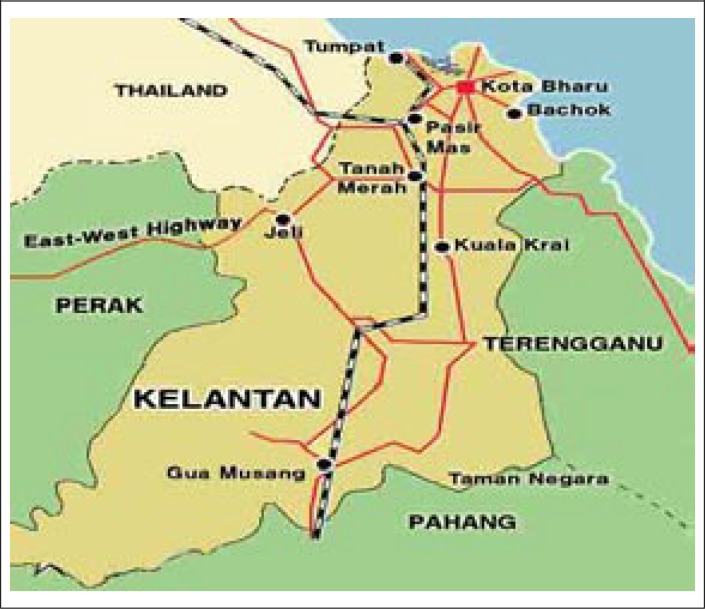
Map of Bachok-Kelantan, Malaysia


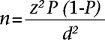

where z=1.96 at 5% level of significance,

*P*=Anticipated population proportion, and

*d*=Absolute precision

Owing to the strict inclusion criteria of households, mothers, and children, non-probability sampling was conducted, and all the respondents were selected from the records of the Social Welfare Department until the calculated sample-size was reached. The calculated sample-size in our study was 203, to which we added 10% suspecting non-response rate. Therefore, the final sample-size was 223 households.

### Data collection

Prior to data collection, permission to carry out the study was obtained from the Social Welfare Department of Malaysia, and the research protocol was approved by the Medical Research Ethical Committee of the Universiti Sains Malaysia. Data collection was carried out using the following instruments:

#### Questionnaire

Data collection from the field was conducted by two trained research assistants (interviewers) who made a home-visit to collect all of the pertinent research information through face-to-face individual interviews with the mother. A pre-tested questionnaire was used for the interview to collect information about household food security. The questionnaire contained items about household demographic and socioeconomic characteristics. The mother was asked about income, household-size, number of children, children's data (age, gender, and educational level), parental age, education, occupation, and quality of life. In some cases, the father was interviewed when the needed information could not be obtained from the mother. To evaluate household food security, the Radimer/Cornell hunger and food insecurity instrument was used. The household food insecurity construct consisted of four components: food quantity, food quality, food acceptability, and certainty of obtaining food. According to the Radimer/Cornell Scale, as food problems worsen, food-related uncertainty and anxiety are first experienced at the household level (mild food insecurity), followed by adult food insecurity (moderate food insecurity) characterized by a decrease in the quality and quantity of food consumed by the adults (3). Child hunger (severe food insecurity) is the most severe household food security problem, and is characterized by a decrease in the quantity of food consumed by the children. Child hunger occurs only after the adults in the household and the quality of the children's diet have been affected by household food insufficiency. The instrument rationalizes that children are the last ones to go hungry in food-insecure households. Based on Radimer/Cornell Scale data obtained from the mothers, children were classified into four mutually-exclusive categories: food-secure, household food-insecure, adult food-insecure, and child hunger (3). The Radimer/Cornell instrument has been shown to be applicable in Malaysia as a direct assessment of household food insecurity (39-41).

#### Anthropometric measurements

The subject's (mother's) height was measured following standard procedures of the World Health Organization (42), using a portable SECA body-meter with a horizontal head-board attachment. Participants removed their shoes and stood as tall and straight as possible with their head level and their shoulders and upper arms relaxed. The vertical distance between the standing surface and the top of the subject's head was measured at the maximum point of inhalation. The measurement was repeated twice to a precision of 0.1 cm, and a mean value calculated. Weight was obtained using a SECA digital weighing scale (to the nearest 0.1 kg). Participants removed their shoes, socks, and all bulky clothing items. The average of two measurements was used in analyses. Body mass index (BMI) was computed using the formula: weight (kg)/height (m)^2^. Waist-circumference (WC) was measured using a flexible tape. Clothe-pins were used for securing clothing for access to the abdominal area. Participants stood straight and relaxed with their arms by their sides and their feet together. The superior border of the iliac crest and the inferior border of the last rib were marked on both lateral sides of the abdomen. The tape was looped around the waist at the midpoint between the two marks. Two consecutive measurements were taken at the anterio-lateral side of the participant at the end of a normal exhalation. WC was recorded with a precision of 0.1 cm, and a mean value was calculated. Research assistants who took the measurements had been trained according to the WHO protocols (28).

### Data analysis

The data were analyzed using the PASW 18.0 program. Descriptive statistics were used in measuring the prevalence of household food insecurity. Households experiencing household and individual food insecurity as well as child hunger were categorized as food-insecure households. Pearson's chi-square analysis and independent *t*-tests were used in comparing categorical and continuous variables between the food-secure and food-insecure households respectively. The odds ratio (OR) for each independent variable (demographic, socioeconomic) with outcome variable (food insecurity) was determined through univariate logistic regression to identify the individual risk factor of food insecurity. All independent variables with a p value <0.25 were considered important factors and included in the multivariable analysis. Significance level was set at p<0.05.

## RESULTS

### Food security status

Table 1 shows the prevalence of food insecurity among the households evaluated in this study. Households were assigned to mutually-exclusive groups representing increasingly severe food insecurity problems. Approximately 83.9% of caretaker respondents revealed that they and the members of their households had experienced periods of food insecurity in the 12 months prior to the interview, with 29.6% (n=66) reporting household food insecurity, 19.3% (n=43) reporting individual food insecurity, and 35.0% (n=78) reporting child hunger.

### Socioeconomic characteristics

The respondents’ mean age was 42.24±6.42 years (range: 22–54 years); 60.5% were aged 31–45 years. Almost half of the mothers (49.3%) attained lower secondary education, and only 8.1% held technical and vocational certificates while 22.0% had low educational level; half of them never had formal schooling.

The average household-size was 6.71±2.29 (range: 2-15). This value was higher than the average household-size of 4.6 reported for households in rural areas of Malaysia (43). More than half (61.4%) of the respondents had a household containing 6–10 people whereas only 8.1% of the families had more than 10 members. Of the children, 95 (42.6%) were male, and 126 (57.4%) were female. The mean±SD age of the children was 91.43±31.46 months (range: 24-143 months). The average number of children per household was 5.14±2.48, and the average number of children going to school per household was 3.05±1.52.

**Table 1.** Prevalence of household food insecurity (n=223)

Food security status	n (%)
Food-secure household	36 (16.1)
Food-insecure household	66 (29.6)
Food-insecure individual	43 (19.3)
Child hunger	78 (35.0)

In 60% of the households, the mother was the single head of the family: 38.1% were widows, and 21.5% were divorced. Sixty-seven percent of the mothers worked outside, and the rest (32.2%) were housewives and had no earning. The mean monthly household income was RM 815.77±365.67. Forty-four percent of the respondents were living with a total income below the poverty line of RM 691, 33.6%, had a total income between RM 691 and RM 1,000, and 22.0% had an income above RM 1,000. Table 2 presents the results of simple and multiple logistic regressions. From the results of simple logistic regression, an independent variable that has a p value <0.25 was considered an important and associated factor and included in the multiple logistic regression analysis. The preliminary final-effect model included only three variables, namely household-size, total income, and food expenditure. The result showed that household-size was statistically associated with food security [adjusted odds ratio (OR_Adj_.) 1.77, 95% CI 1.35-2.32]. The increase in household-size by one member was associated with 77.0% increase in the odds of that household being food-insecure. Our result reported a significant association between total monthly income of the household and food insecurity status, and every decrease in total income of households by 10 RM was associated with 3.0% increase in the odds of being food-insecure (OR _Adj_. 0.997, 95% CI 0.995-0.998). The results showed significant association between total food expenditure and food insecurity, and every decrease in food expenditure by RM 10 was associated with 2.0% increase in the odds of being food-insecure (OR_Adj_. 0.997, 95% CI 0.991-1.000, p=0.049).

### Anthropometric measurements

Table 3 presents the anthropometric data. The average weight and height of the mothers in the study were 59.29±13.71 kg and 151.46±8.0 cm respectively, and the mean BMI and WC value were 25.42±4.96 kg/m2 and 80.12±12.09. Surprisingly, 52% of the mothers were overweight or obese, and only 6.3% were underweight. The result also showed that 47.1% of the mothers had at-risk WC (≥80 cm). Although the mean±SD weight of 59.53±13.6 kg, BMI of 25.44±4.88, and WC of 80.40±12.0 cm among food-insecure mothers were greater than their counterparts in the food-secure households (58.0±13.9, 25.33±5.4, and 78.6±12.5 respectively) the mean differences between the two types of households were not significant for the three measurements. Moreover, the chi-square test emphasized that there was no significant difference in proportions of the WC categories between the mothers in the food-secure and food-insecure households.

**Table 2.** Factors associated with food security status from simple and multiple logistic regression analysis (n=223)

Variable	Simple logistic regression			Multiple logistic regression		
	*b* (SE)	Crude OR (95% CI)	p value	Adjusted *b* (SE)	Adjusted OR (95% CI)	p value*^a^*
Age of mother (years)	-0.003 (0.03)	1.00 (0.94-1.06)	0.920			
Education of mother						
Lower than secondary	0.64 (0.51)	1.90 (0.70-5.20)	0.207			
Secondary and higher	-	1.00	-			
Household-size	0.22 (0.09)	1.24 (1.02-1.49)	0.024	0.57 (0)	1.77 (1.35-2.32)	<0.001
No. of children per household	0.19 (0.08)	1.21 (1.02-1.42)	0.024			
No. of children going to school	0.26 (0.13)	1.29 (1.00-1.66)	0.044			
Type of household						
Single-headed household	0.065 (0.37)	1.06 (0.5-1 2.20)	0.861			
Double-headed household (Ref.)	-	1.00	-			
Employment status						
Housewife	0.25 (0.40)	1.29 (0.58-2.84)	0.528			
Working women (Ref.)	-	1.00	-			
Sex of the child						
Male	0.22 (0.37)	0.80 (0.391-1.637)	0.541			
Female		1.00				
Household income (RM)	-0.002 (0.0)	0.998 (0.997-0.999)	<0.001	-0.003 (0.001)	0.997 (0.995-0.998)	<0.001
Household income per capita^b^	-0.02 (0.003)	0.980 (0.973-9.87)	<0.001			
No. of individuals contributed in household income	-0.57 (0.25)	0.564 (0.345-0.922)	0.022			
Total food expenditure	-0.002 (0.001)	0.998 (0.997-1.00)	0.024	-0.002 (0.001)	0.997 (0.991-1.000)	0.049

^a^Significance level p<0.05;

^b^Income per capita was not included in the multiple logistic regression

**Table 3.** Food security status and nutritional status of mother (n=223)

Variable	Total	Food-secure (n=36)		Food-insecure (n=187)		Mean difference (95% CI)/*χ^2^*	p valuea
		n (%)	Mean±SD	n (%)	Mean±SD		
Weight (kg)	59.29±13.71		58.06±13.93		59.53±13.62	-1.48 (-6.40,3.44)	0.554^†^
Height (cm)	151.91±5.46		151.1±4.30		152.06±5.62	-0.92 (-3.26,2.49)	0.358^†^
BMI (kg/m^2^)	25.42±4.96		25.33±5.40		25.44±4.88	-0.11 (-1.90,1.66)	0.896^†^
BMI category						2.11 (3.0)	0.550^¶^
<18.5	14 (6.3)	2 (5.6)		12 (6.4)			
18.5-<25	93 (41.7)	18 (50.0)		75 (40.1)			
25-<30	78 (35.0)	9 (25.0)		69 (36.9)			
≥30	38 (17.0)	7 (19.4)		31 (16.6)			
Waist-circumference (cm)	80.12±12.09		78.66±12.5		80.40±2.0	-1.71 (-6.05, 2.63)	0.438^†^
Waist-circumference classificationsb						3.25 (1.0)	0.100^¶^
<80 cm	118 (52.9)	24 (66.6)		94 (50.2)			
≥80 cm	105 (47.1)	12 (33.3)		93 (49.7)			

†Independent *t*-test; ¶Pearson's chi-square; aSignificant at p<0.05, ^b^(WHO, 1998)

## DISCUSSION

Out of the 223 households, 187 (83.9%) reported certain levels of food insecurity, with 66 (29.6%), 43 (19.3%), and 78 (35%) categorized under food-insecure households, households with food-insecure adults, and households with child hunger respectively. The prevalence of household food insecurity in this research is, to some extent, consistent with that in a previous study (77.5%) conducted in the neighbouring district of Tumpat (39). However, previous studies on low-income households in Malaysia indicated that the prevalence of overall food insecurity (58.0% and 65.6%) (40,41) was lower than our findings (83.1%) in Bachok, Kelantan. The higher proportions in the household food insecurity reported in this study could be attributed to the higher rate of poor households in this sample (44.4%), which is substantially higher than that in Malaysia (5.7%) and in Kelantan (10.6%) based on poverty line income (44). Hence, the variations were not surprising. Moreover, the respondents of this study were selected purposively based on several specific and strict criteria, such as being recipients of financial aid from the Social Welfare Department because of socioeconomic constraints. With limited funding and fewer economic opportunities, the number of households living below poverty line in the state has swelled to well over 3,000, and nearly half of these (1,450 families) are from Bachok district (45), which is another important factor that may be attributed to the increased risk of food insecurity in Bachok district. The non-significant association between educational level and household food insecurity might be explained by the fact that majority of our respondents were moderately educated and that the educational level was almost distributed equally between the two types of households. Any increase in the educational level among the mothers will not decrease the risk of the household being food-insecure because educational level did not have any significance when the family was under economical constraints. Another explanation for the findings is the Malaysian Government's efforts to bridge the educational gap between the urban and poor rural areas (from 2001 to 2005, the Government allocated RM 43.7 billion for education and training) (46). Apart from that, larger households were more likely to be food-insecure than small households (41,47). However, marital status of the mother was not associated with household food insecurity. The result opposed Radimer's finding which emphasized that higher prevalence of food insecurity was associated with the mother's marital status, whether single or separated, divorced, widowed, or married (48) while the result was consistent with Hanson's finding, wherein widowed women experienced relatively high levels of food security (49). This study found no association between household food insecurity level and employment status of the mother, contrary to previously-existing evidence (50,51). Generally, working mothers are expected to have better access to food and food security conditions. However, others argued that food security status was negatively affected when mothers worked outside the home (52). The study showed a negative association between the total household income and household food insecurity status. Therefore, an increase in the household monthly income by RM 10 will decrease the odds of being food-insecure by 3%. Likewise, the more money the household has, the more access it has to better food in terms of quality or quantity. The relationship between income and household food security is a sequential relationship between food expenditure and diet diversity that leads to food security (29). The findings are consistent with previous studies which reported that household income influences household food security status. In brief, those households with lower incomes are at risk of food insecurity (3,51,53,54). This absence of relationship between the number of household members contributing to the household income and household food insecurity status can be attributed to the negligible financial contributions of the other working members because they work part time. The relatively high combined income of two persons in the same household does not necessarily guarantee the allocation of adequate money for food (41). Income and food security measures refer to all the persons living at the same address. In some cases, these persons comprise more than one economic unit, with little or no sharing of income with the primary household. Unusually-high economic needs, such as severe medical conditions, addictions, and other contingencies deplete the income of households and can reduce the money allocated for food (55). The present study indicated how total food expenditure is associated with food insecurity. Food-secure households with higher income will be able to provide better nutrition for their children because they have more purchasing power, allowing them to choose and prepare healthful but more expensive foods (56). This situation might explain the intricate relationship among food security, food expenditure, and diet diversity.

The results of the present study do not support the hypothesis that food insecurity and obesity are associated. In contrast, several cross-sectional studies have found that obesity is more common in women who report food insecurity or insufficiency than in those who do not (23,24,57,58). However, other studies have not found this association in women (59,60), and some that found it in women did not find it in men (25,58). Although the relationship between overweight and poverty seems paradoxical, the current study reported higher rates of overweight and obesity in the targeted poor rural Malaysian households. The difference between the distributions of overweight and obesity into the two types of households was not large and was almost homogenous. The absence of the association between household food insecurity status and maternal overweight and obesity can be explained in different ways. Cheap prices of energy-dense foods and financial constraints may encourage overconsumption of an energy-rich but low-quality diet, leading to weight gain for individuals in poor communities (23,59,61-63). In addition, because the majority of food-poor households receive assistance from one or more federal food assistance programmes, such as Food Basket Program and School Milk Program implemented by the Malaysian Government to eradicate poverty and malnutrition among poor Malaysian (64), the mothers may indulge in over-eating when food stamps are available, followed by a short period of involuntary food deprivation. This continuous cycle of temporary food abundance and deprivation may result in gradual weight gain (25). Our findings were consistent with those of Jones who suggested that food insecurity at one time point is not strongly or consistently associated with women's subsequent weight gain (30). In fact, obesity is a chronic condition which develops over a period of months to years, and food insecurity can be periodic or chronic. Because individuals experience these conditions over time, their association cannot necessarily be explained by a study in which each is measured at only one or two points in time. There may be a long lag in the impacts of food security on weight gain and obesity (31). The selection of a food insecurity instrument is a key factor that has been shown to influence the detection of the association between food insecurity status and obesity. Kaiser and colleagues reported the relationship between food insecurity and obesity, using the 18-item US Household Food Security Scale. This association was absent, however, when the current status of food insecurity was measured using a single-item questionnaire (65).

### Limitations

In this study, the sample-size was small and restricted to subjects who received a monthly welfare allowance. As such, the records of the Social Welfare Department might have underrepresented the poor families in Bachok district for various reasons. Conversely, some of the families included in these records generated new incomes, which brought them out of the poverty circle. Despite these sampling issues, our data indicate that food insecurity may represent an appreciable problem in low-income rural households in Malaysia. Household food insecurity was highly prevalent in low-income households in the study area of rural Malaysia despite government efforts to tackle the problem. In cross-sectional studies, one-point time measurement is not an appropriate method for judging the association between household food insecurity and nutritional status of the mother; hence, multiple measurements in longitudinal studies would allow investigators to capture transitions and turning points in the association.

### Conclusions

In this cross-sectional study of poor households in Bachok, a correlation between food insecurity and obesity was not detected. Overweight and obesity require a long period of time to develop, and over time, households may have survived waves of periodic food insecurity. These temporal factors limit the usefulness of snapshot studies. To trace the association between food insecurity and weight gain in adults, longitudinal studies with larger sample-sizes are recommended.

## ACKNOWLEDGEMENTS

This work was supported by a research grant (No. 1001/PPSK/812022) from the Universiti Sains Malaysia. The authors would like to express their gratitude and appreciation to all the participants and staff and to Miss Fiona Lim Wei Ting and Mr. Azizi bin Mohamed Zain who assisted in this study. Profound appreciation is extended to the Social Welfare Department of Malaysia for giving permission to conduct the study. The authors would also like to thank statistician Dr. Kamarul Imran for his help and guidance in data analysis.
